# Supplementary material for: Quenching protein dynamics interferes with HIV capsid maturation
Source: Nat Commun. 2017 Nov 24;8:1779. doi: 10.1038/s41467-017-01856-y (PMC5701193; doi:10.1038/s41467-017-01856-y)
Supplement: Supplementary file 1 — Supplementary Information [file 41467_2017_1856_MOESM1_ESM.pdf]

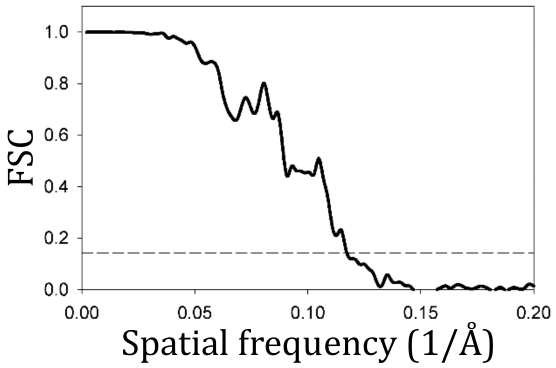

Figure 1. CryoEM reconstruction of CA-SP1 assemblies: Fourier Shell Correlation (FSC) plot of 3D density map. The map resolution is 8.4 Å at FSC of 0.143.

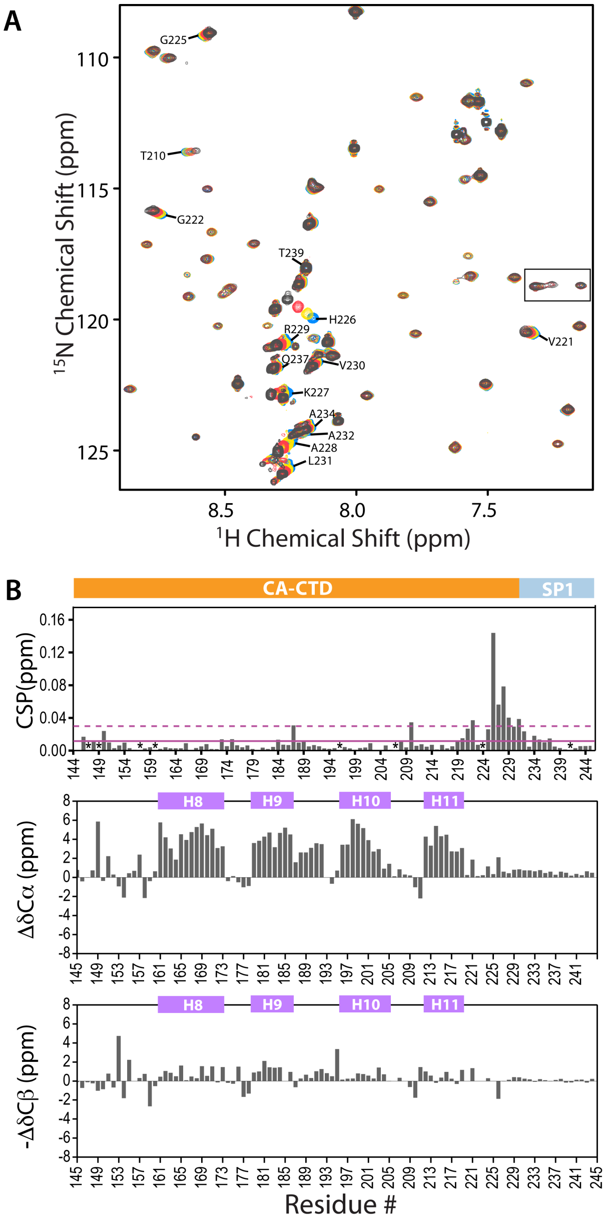

Figure 2. DFH-055 methanesulfonate binding to CA-CTD-SP1 (A) Superposition of a selective region of the ^{1}H-^{15}N HSQC spectra of 1.11 mM CA-CTD-SP1 in the absence (blue) and presence of 1.11 mM (yellow), 2.22 mM (red) and 3.33 mM (black) DFH-055 methanesulfonate at 298K, 800 MHz. Representative CA-CTD-SP1 resonances are labeled with residue names and numbers. Folded arginine side chain Nε resonances are enclosed in the rectangle. (B) Top: ^{1}H,^{15}N-combined chemical shift changes, plotted along the linear amino acid sequence, were calculated using the equation $(\Delta\delta_{HN}^{2} + (0.15*\Delta\delta_{N})^{2})^{1/2}$, with $\Delta\delta_{HN}$ and $\Delta\delta_{N}$ representing ^{1}HN and ^{15}N chemical shift differences between CA-CTD-SP1 resonances in the absence and presence (3.33 mM) of DFH-055 methanesulfonate, respectively. The solid and dashed horizontal lines indicate the average chemical shift change (0.011 p.p.m.) and the sum (0.030 p.p.m) of the average change plus one S.D., respectively. Proline residues and the unassigned S149 positions are marked with *. Middle and bottom panels: Secondary chemical shifts (ΔC_{α}, middle; and $-\Delta C_{\beta}$, bottom) of CA-CTD-SP1 in solution. ΔC_{α} and ΔC_{β} were calculated by subtracting random coil C_{α} and C_{β} shifts[1] from the measured values.

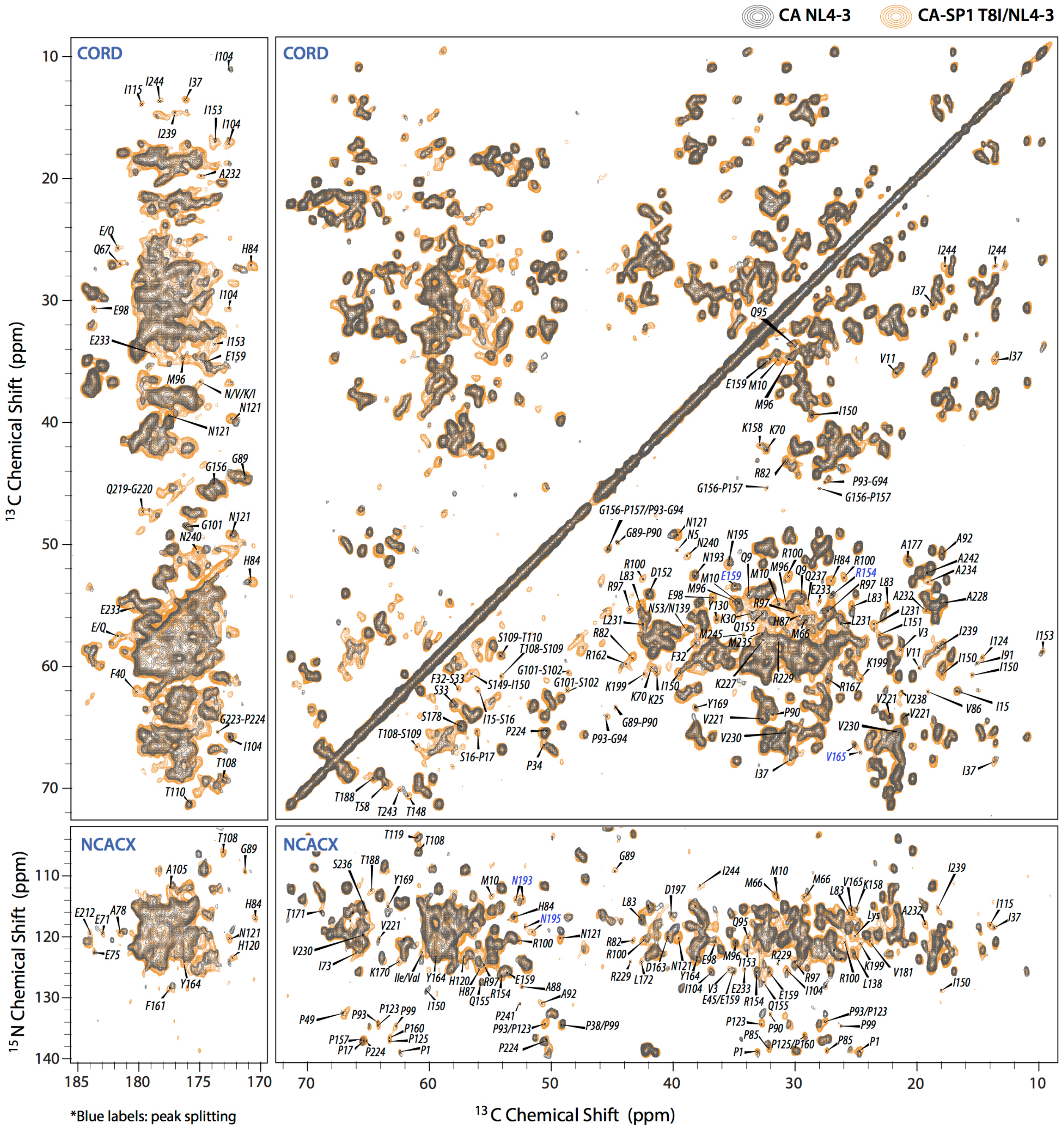

Figure 3. Comparison of dipolar-based spectra of HIV-1 CA and CA-SP1 T8I tubular assemblies. 2D CORD (top) and NCACX (bottom) MAS NMR spectra for CA NL4-3 (gray) and CA-SP1 NL4-3 T8I (orange), illustrating the multiple chemical shift and peak intensity changes. Residues for which resonance doubling is observed are labeled in blue. All spectra were acquired at 20.0 T with a MAS frequency of 14 kHz, and processed with 60° shifted sine bell apodization in both dimensions.

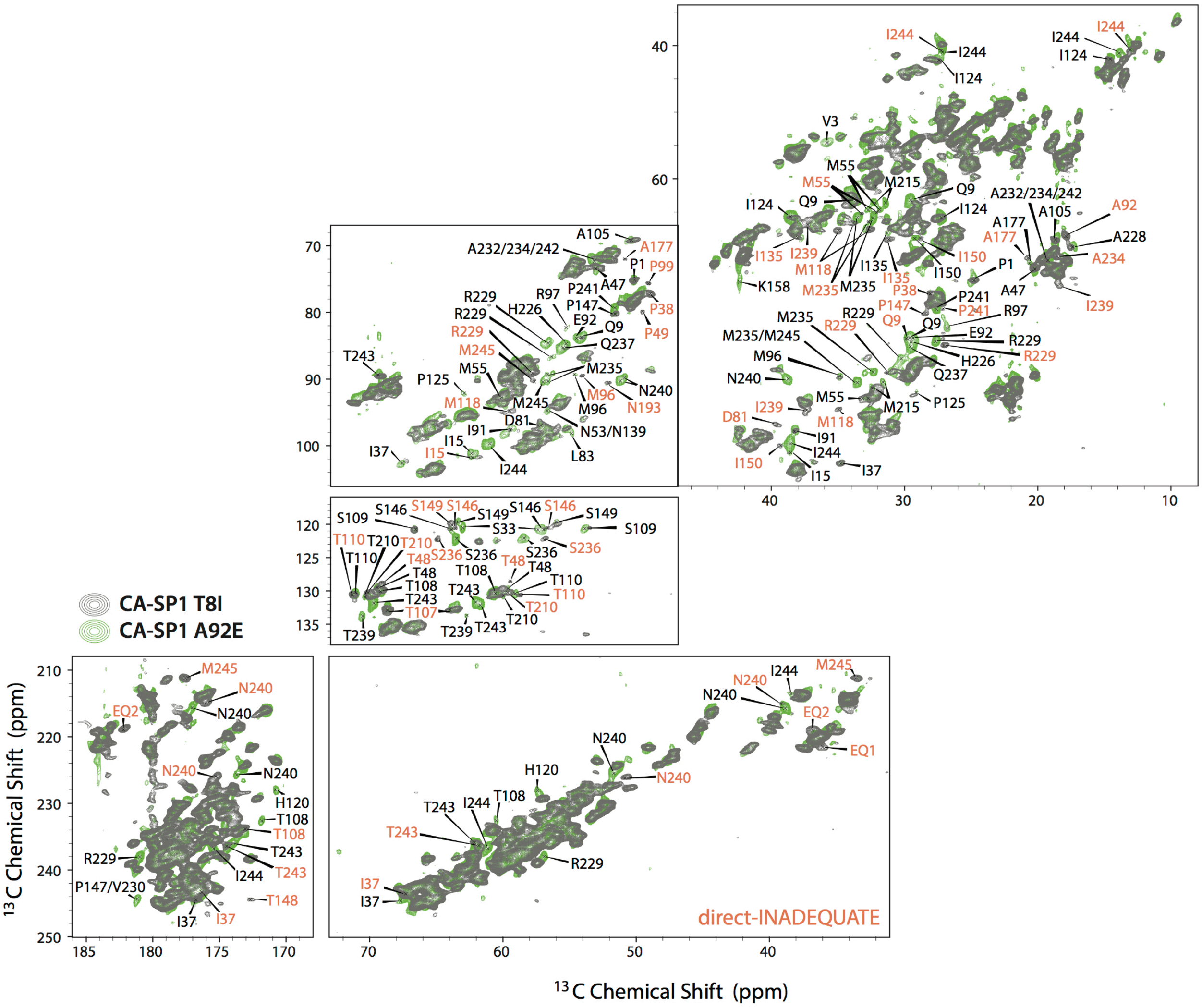

Figure 4. Comparison of J-based spectra for different HIV-1 CA-SP1 variants. 2D direct-INADEQUATE MAS NMR spectra for CA-SP1 NL4-3 A92E (green, black labels) and CA-SP1 NL4-3 T8I (gray, orange labels), illustrating numerous chemical shift changes associated with residues of CypA loop and CTD (V221-) SP1 tail. Note the reduced number of signals in the spectrum of CA-SP1 T8I mutant compared to the equivalent protein carrying a WT SP1 tail, demonstration the reduced dynamics in the SP1 T8I tubular assemblies. The spectra of CA-SP1 A92E assemblies were acquired at 20.0 T, and the spectra of CA-SP1 A92E assemblies were recorded at 14.4 T, both with a MAS frequency of 14 kHz. They were processed with 60° shifted sine bell apodization in both dimensions.

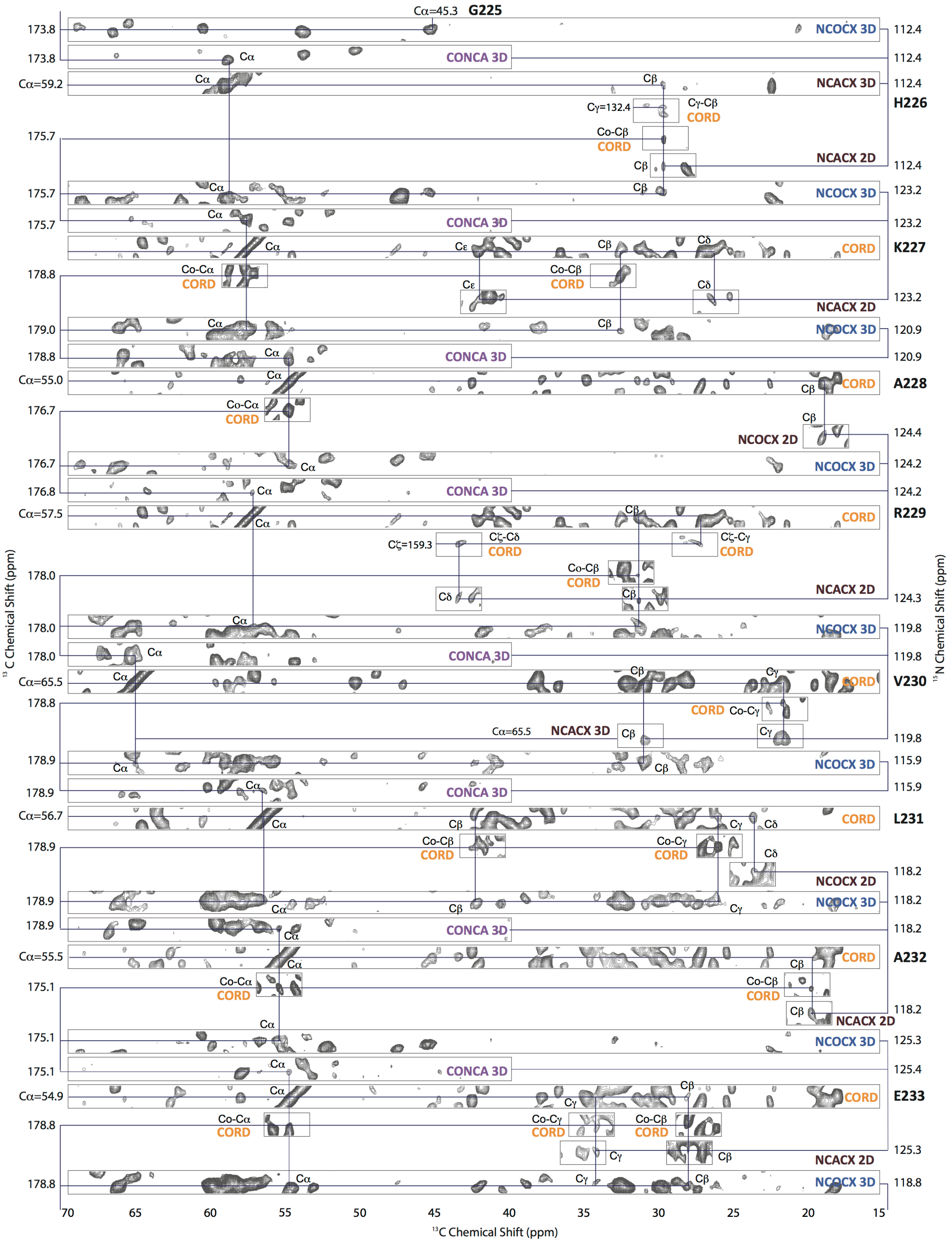

Cα=45.3
G225
173.8
NCOCX 3D
112.4
173.8
Cα
CONCA 3D
112.4
Cα=59.2
Cα
Cβ
NCACX 3D
112.4
H226
Cγ=132.4
Cγ-Cβ
CORD
175.7
Co-Cβ
CORD
Cβ
NCACX 2D
112.4
175.7
Cα
Cβ
NCOCX 3D
123.2
175.7
Cα
CONCA 3D
123.2
Cα
Cε
Cβ
Cδ
CORD
K227
178.8
Co-Cα
CORD
Co-Cβ
CORD
Cε
Cδ
123.2
NCACX 2D
179.0
Cα
Cβ
NCOCX 3D
120.9
178.8
Cα
CONCA 3D
120.9
Cα=55.0
Cα
Cβ
CORD
A228
176.7
Co-Cα
CORD
Cβ
NCOCX 2D
124.4
176.7
Cα
NCOCX 3D
124.2
176.8
Cα
CONCA 3D
124.2
Cα=57.5
Cα
Cβ
CORD
R229
Cζ=159.3
Cζ-Cδ
CORD
Cζ-Cγ
CORD
178.0
Co-Cβ
CORD
NCACX 2D
Cδ
Cβ
124.3
178.0
Cα
NCOCX 3D
119.8
178.0
Cα
CONCA 3D
119.8
Cα=65.5
Cα
Cβ
Cγ
CORD
V230
178.8
CORD
Co-Cγ
Cα=65.5
NCACX 3D
Cβ
Cγ
119.8
178.9
Cα
Cβ
NCOCX 3D
115.9
178.9
Cα
CONCA 3D
115.9
Cα=56.7
Cα
Cβ
Cγ
Cδ
CORD
L231
178.9
Co-Cβ
CORD
Co-Cγ
CORD
Cδ
118.2
NCACX 2D
178.9
Cα
Cβ
Cγ
NCOCX 3D
118.2
178.9
Cα
CONCA 3D
118.2
Cα=55.5
Cα
Cβ
CORD
A232
175.1
Co-Cα
CORD
Co-Cβ
CORD
Cβ
NCACX 2D
118.2
175.1
Cα
NCOCX 3D
125.3
175.1
Cα
CONCA 3D
125.4
Cα=54.9
Cα
Cγ
Cβ
CORD
E233
178.8
Co-Cα
CORD
Co-Cγ
CORD
Co-Cβ
CORD
Cγ
Cβ
125.3
NCACX 2D
178.8
Cα
Cγ
Cβ
NCOCX 3D
118.8
70
65
60
55
50
45
40
35
30
25
20
15
^{13}C Chemical Shift (ppm)
^{13}C Chemical Shift (ppm)
^{15}N Chemical Shift (ppm)

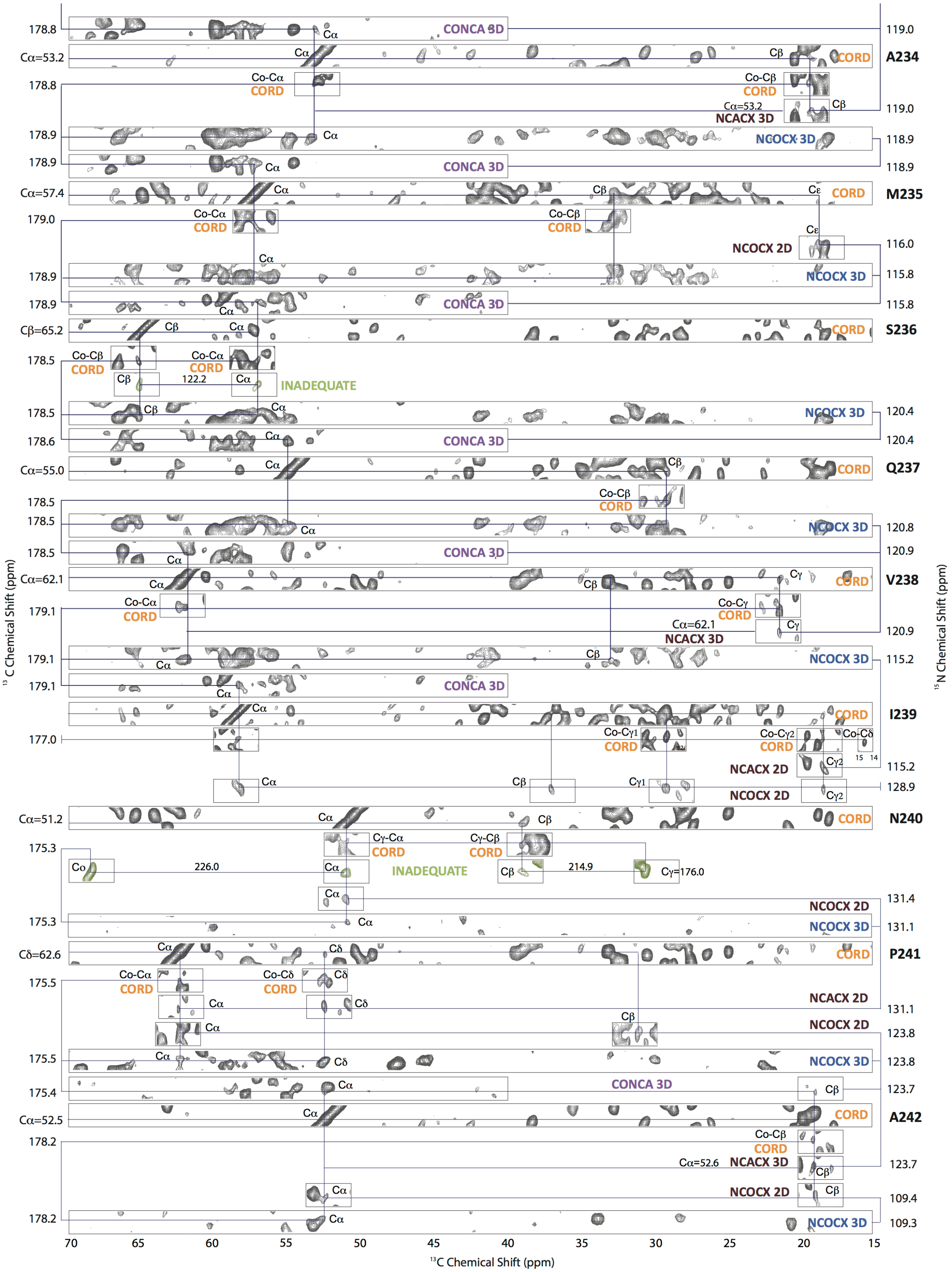

178.8
Cα
CONCA 3D
119.0
Cα=53.2
Cα
Cβ
CORD
A234
178.8
Co-Cα
CORD
Co-Cβ
CORD
Cα=53.2
NCACX 3D
Cβ
119.0
178.9
Cα
NCOCX 3D
118.9
178.9
Cα
CONCA 3D
118.9
Cα=57.4
Cα
Cβ
Cε
CORD
M235
179.0
Co-Cα
CORD
Co-Cβ
CORD
Cε
NCOCX 2D
116.0
Cα
178.9
NCOCX 3D
115.8
178.9
Cα
CONCA 3D
115.8
Cβ=65.2
Cβ
Cα
CORD
S236
178.5
Co-Cβ
CORD
Co-Cα
CORD
Cβ
122.2
Cα
INADEQUATE
178.5
Cβ
Cα
NCOCX 3D
120.4
178.6
Cα
CONCA 3D
120.4
Cα=55.0
Cα
Cβ
CORD
Q237
Co-Cβ
CORD
178.5
178.5
Cα
NCOCX 3D
120.8
178.5
CONCA 3D
120.9
Cα
Cα=62.1
Cα
Cβ
Cγ
CORD
V238
179.1
Co-Cα
CORD
Co-Cγ
CORD
Cα=62.1
NCACX 3D
Cγ
120.9
179.1
Cα
Cβ
NCOCX 3D
115.2
179.1
Cα
CONCA 3D
Cα
CORD
I239
177.0
Co-Cγ1
CORD
Co-Cγ2
CORD
Co-Cδ
Cγ2
15
14
NCACX 2D
115.2
Cα
Cβ
Cγ1
NCOCX 2D
Cγ2
128.9
Cα=51.2
Cα
Cβ
CORD
N240
175.3
Cγ-Cα
CORD
Cγ-Cβ
CORD
Co
226.0
Cα
INADEQUATE
Cβ
214.9
Cγ=176.0
Cα
NCOCX 2D
131.4
175.3
Cα
NCOCX 3D
131.1
Cδ=62.6
Cα
Cδ
CORD
P241
175.5
Co-Cα
CORD
Co-Cδ
CORD
Cδ
Cα
Cδ
NCACX 2D
131.1
Cα
Cβ
NCOCX 2D
123.8
175.5
Cα
Cδ
NCOCX 3D
123.8
175.4
Cα
CONCA 3D
Cβ
123.7
Cα=52.5
Cα
CORD
A242
178.2
Co-Cβ
CORD
Cα=52.6
NCACX 3D
Cβ
123.7
Cα
NCOCX 2D
Cβ
109.4
178.2
Cα
NCOCX 3D
109.3
70
65
60
55
50
45
40
35
30
25
20
15
13C Chemical Shift (ppm)
13 C Chemical Shift (ppm)
15N Chemical Shift (ppm)

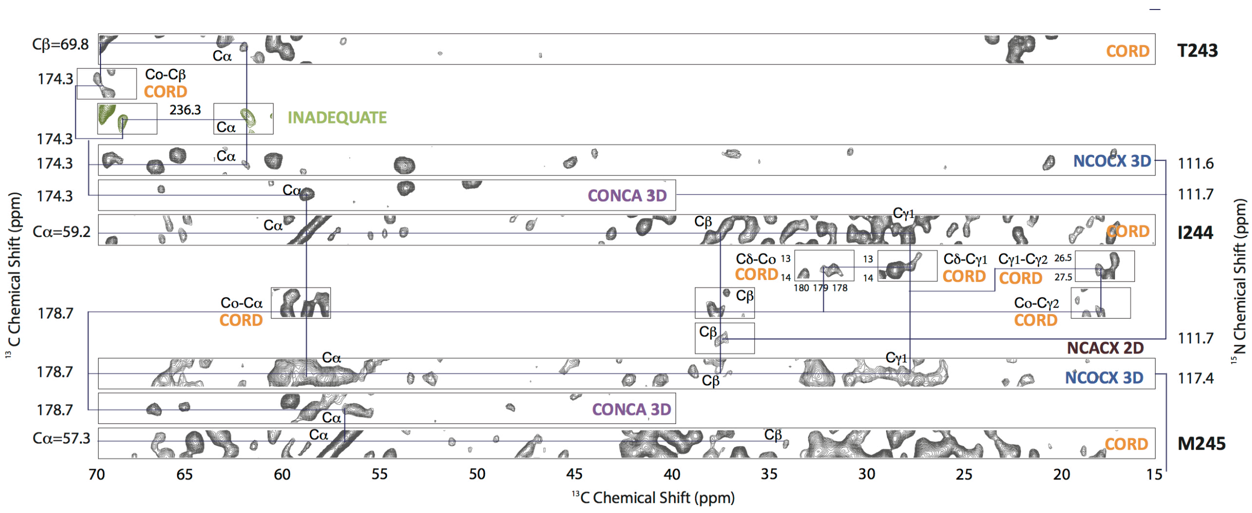

Figure 5. Sequential walk through the CTD-SP1 region for residues V221 to M245 in CA-SP1 NL4-3 T8I tubular assemblies. Resonances for this stretch of residues were assigned by a combination of several types of MAS NMR spectra: 2D CORD (orange labels), 2D/3D(NUS) NCACX (brown labels), 2D/3D NCOCX (brown / blue labels, respectively), 3D CONCA (purple labels), and 2D direct-INADEQUATE (green labels).

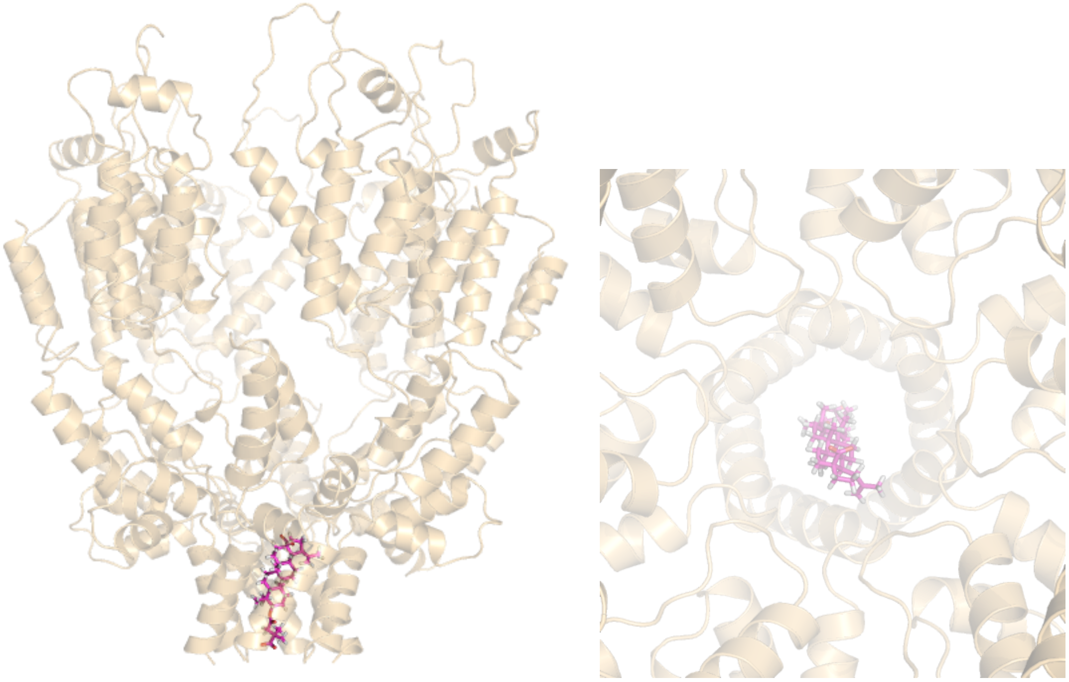

Figure 6. A binding pose of BVM (magenta) in the CTD-SP1 hexamer (X-ray structure PDB code 5L93) resulting from rigid body docking. Left: view from the side; right: view from the top.

Table 1. Experimental MAS NMR chemical shifts for the CTD-SP1 region (residues 221-245) of CA-SP1 T8I assemblies.

Residue	N	CO	CA	CB	CG	CD	CE	CZ	CSI 2.0
V221	120.53	176.98	64.29	32.72	22.18/21.04	-	-	-	C
G222	117.30	173.75	45.13	-	-	-	-	-	C
G223	112.59	173.82	45.23	-	-	-	-	-	C
P224	137.09	177.61	65.34	32.38	28.05	50.41	-	-	C
G225	116.27	173.80	45.35	-	-	-	-	-	C
H226	112.55	175.70	59.31	29.88	132.41	-	-	-	H
K227	123.19	179.00	57.90	32.59	26.16	29.89	42.09	-	H
A228	120.89	176.71	54.88	18.09	-	-	-	-	H
R229	124.17	177.96	57.54	31.37	27.10	43.63	-	159.28	H
V230	119.79	178.86	65.47	31.27	21.72	-	-	-	H
L231	115.90	178.92	56.83	42.34	26.23	23.47	-	-	H
A232	118.24	175.09	55.70	19.55	-	-	-	-	H
E233	125.35	178.80	54.89	28.09	34.11	-	-	-	H
A234	118.88	178.90	53.14	19.10	-	-	-	-	H
M235	118.89	179.03	57.42	32.54	-	-	18.64	-	H
S236	115.76	178.53	57.05	65.20	-	-	-	-	H
Q237	120.47	178.52	54.99	29.04	-	-	-	-	H
V238	120.50	179.06	62.10	33.09	21.22		-	-	C
I239	115.29	177.05	58.52	36.85	29.17/18.26	14.6	-	-	C
N240	128.80	175.30	51.10	39.13	176.00		-	-	C
P241	131.10	175.52	62.57	31.21	28.78	52.57	-	-	C
A242	123.70	178.19	52.54	18.92	-	-	-	-	C
T243	109.60	174.40	62.20	69.82	-	-	-	-	C
I244	111.60	178.65	59.22	37.75	27.59/17.87	13.77			C
M245	117.40		57.33	34.16/33.69	-	-	-	-	C

Table 2. Secondary structure predictions for the CTD-SP1 region (residues 221-245) determined by MAS NMR for different CA-SP1 variants. CSI 2.0[2] and TALOS-N [3] programs were used to predict secondary structures from the chemical shift index (CSI) and torsion angles, respectively. Designations C-coil and H-helix are as described in Materials and Methods.

	**NL4-3/T8I**		**NL4-3/A92E**		**HXB2**		**NL4-3/solution NMR**	
**Residue**	**CSI 2.0**	**TALOS-N**	**CSI 2.0**	**TALOS-N**	**CSI 2.0**	**TALOS-N**	**CSI 2.0**	**TALOS-N**
V221	C	C	C	C	C	C	C	C
G222	C	C	C	C	C	C	C	C
G223	C	C	C	C	C	C	C	C
P224	C	C	-	-	-	C	C	C
G225	C	C	-	-	-	C	C	C
H226	H	H	C	-	C	C	C	C
K227	H	H	C	C	C	-	C	C
A228	H	H	C	-	-	-	C	C
R229	H	H	C	H	C	-	C	C
V230	H	H	H	H	-	-	C	C
L231	H	H	H	H	C	-	C	C
A232	H	H	H	H	H	-	C	C
E233	H	H	H	H	H	H	C	H
A234	H	H	C	-	H	-	C	H
M235	H	H	C	-	-	-	C	C
S236	H	H	C	-	-	-	C	C
Q237	H	C	C	-	-	-	C	C
V238	C	C	-	-	-	-	C	C
I239/T239	C	C	C	-	C	-	C	C
N240	C	C	C	-	-	-	C	C
P241	C	C	-	-	-	-	H	C
A242	C	C	C	-	C	-	H	C
T243	C	C	C	-	-	-	H	C
I244	C	C	C	-	-	-	C	C
M245	C	C	C	-	-	-	C	C

"-" The predictions are less reliable due to the missing chemical shifts.

Table 3. Secondary structure predictions for the NTD in CA-SP1 assemblies by MAS. CSI 2.0[2] and TALOS-N[3] programs were used to predict secondary structures from the chemical shift index (CSI) and torsion angles, respectively.

	**NL4-3/T8I**		**NL4-3/A92E**		**HXB2**	
**Residue**	**CSI 2.0**	**TALOS-N**	**CSI 2.0**	**TALOS-N**	**CSI 2.0**	**TALOS-N**
P1	C	C	C	-	C	C
I2	B	C	B	-	B	C
V3	B	B	B	B	B	B
Q4	B	B	C	B	C	B
N5	C	C	C	C	C	C
L6/I6	H	H	C	C	C	H
Q7	C	H	C	H	C	H
G8	C	H	H	H	C	H
Q9	C	C	C	C	C	C
M10	C	B	B	B	B	B
V11	B	B	B	B	B	B
H12	B	B	B	B	B	B
Q13	B	B	B	B	B	B
A14	C	B	B	B	B	B
I15	C	C	C	C	C	C
S16	C	C	C	C	C	C
P17	H	H	H	H	H	H
R18	H	H	H	H	H	H
T19	H	H	H	H	H	H
L20	H	H	H	H	H	H

"-" The predictions are less reliable due to the missing chemical shifts.

Table 4. Temperature rungs and its associated weights used for the simulated-tempering simulation of wild-type CA-SP1.

Temperature Rung (K)	Simulated Tempering Weight
310.0	-30301.841
313.5	-26789.562
317.0	-23365.365
320.5	-20026.189
324.0	-16769.137
327.5	-13591.374
331.0	-10490.412
334.5	-7463.558
338.0	-4508.334
341.5	-1622.494
345.0	1196.552
348.5	3951.036
352.0	6642.746
355.5	9273.551
359.0	11845.700
362.5	14360.941
366.0	16820.933
369.5	19227.457
373.0	21582.033
376.5	23886.104
380.0	26141.213

Table 5. Temperature rungs and associated weights used in the simulated-tempering simulation (REF) of the CA-SP1(T8I) mutant.

Temperature Rung (K)	Simulated Tempering Weight
310.0	-30503.934
313.5	-26969.450
317.0	-23523.689
320.5	-20163.410
324.0	-16885.355
327.5	-13686.826
331.0	-10565.196
334.5	-7517.995
338.0	-4542.650
341.5	-1636.687
345.0	1201.979
348.5	3975.335
352.0	6685.528
355.5	9335.030
359.0	11925.878
362.5	14459.436
366.0	16937.267
369.5	19361.216
373.0	21732.931
376.5	24054.204
380.0	26326.389

REFERENCES

1 Wishart, D. S., Bigam, C. G., Holm, A., Hodges, R. S. & Sykes, B. D. ^{1}H, ^{13}C and ^{15}N random coil NMR chemical shifts of the common amino acids. I. Investigations of nearest-neighbor effects. *J Biomol NMR* **5**, 67-81, doi:10.1007/bf00227471 (1995).

2 Hafsa, N. E. & Wishart, D. S. CSI 2.0: a significantly improved version of the Chemical Shift Index. *J Biomol NMR* **60**, 131-146, doi:10.1007/s10858-014-9863-x (2014).

3 Shen, Y. & Bax, A. Protein Backbone and Sidechain Torsion Angles Predicted from NMR Chemical Shifts using Artificial Neural Networks. *J Biomol NMR* **56**, 227-241, doi:10.1007/s10858-013-9741-y (2013).
